# P22 Between the devil and the deep blue sea – a diagnostic dilemma

**DOI:** 10.1093/rap/rkab068.021

**Published:** 2021-10-19

**Authors:** Premila Kadamban, Mohammad Tariq

**Affiliations:** Southend University Hospital, Southend-on-sea, United Kingdom

## Abstract

**Case report - Introduction:**

Uveitis is characterised by inflammation of the uvea, which is the middle portion of the eye; the anterior portion of the uvea includes the iris and ciliary body, and the posterior portion of the uvea is known as the choroid. There are multiple infections and inflammatory diseases that could cause uveitis. The aetiology of uveitis is often very difficult to decipher; especially in the absence of typical extra-ocular symptoms.

**Case report - Case description:**

We report a case of a 41-year-old young Caucasian woman who presented to the eye clinic in February 2019 with severe redness and pain in the left eye. On slit lamb examination she was found to have acute anterior uveitis (AAU); her symptoms completely resolved with topical steroids.

After 10 months she again presented with AAU in the left eye; her symptoms once again improved on topical steroids. On this occasion the ophthalmology team arranged some investigations to find out the aetiology of uveitis. The infective screen was unremarkable apart from borderline positive T-Spot. She was found to be positive for HLA-B27.

Her repeat T-Spot was again borderline positive. She didn’t have any systemic or constitutional symptoms. She had BCG vaccination in the past. There was no contact history with tuberculosis (TB) patients. She was a teacher at a school for children of ethnic diversity. Her CT scan showed radiolucent circular lesions in the spleen which could represent granulomas.

She commenced anti-TB medicines in September 2020.

She was seen in the rheumatology clinic in November 2020 in view of her positive HLA-B27. On further questioning she mentioned about having an on-going lower back pain and alternating buttock pain for nearly 5 years. She denied having any stiffness (please note she exercises regularly in the mornings). There was no history of skin psoriasis or bloody diarrhoea. On examination she had good range of movement in the spine and didn’t have any SIJ tenderness. She didn’t have any features of peripheral articular or enthesial inflammation.

Her CRP had never been elevated; her ACE level was 41 U/L (normal 8 - 52). Her MRI (STIR sequence) showed features compatible with erosive sacroiliitis and spondylitis.

As the patient had completed 3 months of anti-TB treatment it was deemed suitable to commence anti-TNF therapy.

**Case report - Discussion:**

There are multiple infective and inflammatory diseases that could cause uveitis. CMV, toxoplasmosis, syphilis, cat scratch disease, West Nile virus, Ebola, Zika and TB are to name but a few infections that cause uveitis. There are many systemic inflammatory diseases that cause uveitis as well. spondyloarthropathies, sarcoidosis, juvenile idiopathic arthropathies, psoroaitic arthritis, multiple sclerosis, Behcet’s disease, relapsing polychondritis, and Kawasaki’s disease are few of them.

Nearly 20—30% of patients with axial spondyloarthropathies tend to have acute anterior uveitis. It iis generally unilateral and acute i.e., it resolves in 3 months even without treatment. Uveitis in context of psoriatic arthritis and inflammatory bowel disease can be bilateral, chronic and can involve posterior part of the uvea.

Diagnosis of TB uveitis is not an easy process as there are no unique ocular features, standardised diagnostic criteria and reliable laboratory methods. Usually, presence of extra-ocular features of TB, Tuberculin skin test, Quantiferon assay, circumstantial factors like ethnicity and travel and contact history aid in the diagnosis of TB uveitis. Generally, it is important to have high index of suspicion to diagnose TB uveitis.

There are certain ocular features that are highly predicative of TB uveitis. In an observational study performed in India, it was revealed that broad-based posterior synechiae, retinal vasculitis with or without choroiditis, and serpiginous-like choroiditis were seen significantly more commonly in patients with tubercular uveitis. Presence of Busacca’s and Koeppe’s nodules which represent granulomas are highly linked with but not unique to TB uveitis.

**Case report - Key learning points:**

In our particular case, positive T- spot test, positive HLA B27 and other imaging findings pose a diagnostic dilemma between TB and axial spondyloarthropathy. As our patient had a typically unilateral, acute uveitis which resolved within 3 months and only involved anterior uvea, we believe axial spondyloarthropathy is the likely aetiology of uveitis; however, the patient is to finish her anti-TB treatment. In the meantime, she is to commence biologic treatment for axial spondyloarthropathy.

Anti-TNF treatment has been the traditional first-line biologic in axial spondyloarthropathy. TNF alpha is a very important cytokine in the immunity against *Mycobacterium Tuberculosis* as it maintains the integrity of granuloma. Several studies revealed that anti-TNF treatment causes TB reactivation. Typically, monoclonal antibodies (infliximab, adalimumab, golimumab and certolizumab) have higher risk to cause TB reactivation because of their solubility and ability to bind membrane-bound TNF receptors as well.

Etanercept would be favourable as it has the least potency to reactivate TB. Ironically, it has the potential of increasing the risk of recurrent anterior uveitis because of its ability to attach to TNF-beta, which is important for uveal health as proven in animal models.

Therefore, in this case the diagnostic accuracy is imperative as the choice of treatment could cause adverse effects if it was wrong.

